# A novel scalable electrode array and system for non‐invasively assessing gastric function using flexible electronics

**DOI:** 10.1111/nmo.14418

**Published:** 2022-06-14

**Authors:** Armen A. Gharibans, Tommy C. L. Hayes, Daniel A. Carson, Stefan Calder, Chris Varghese, Peng Du, Yaara Yarmut, Stephen Waite, Celia Keane, Jonathan S. T. Woodhead, Christopher N. Andrews, Greg O'Grady

**Affiliations:** ^1^ Department of Surgery University of Auckland Auckland New Zealand; ^2^ Alimetry Ltd Auckland New Zealand; ^3^ Auckland Bioengineering Institute University of Auckland Auckland New Zealand; ^4^ Maurice Wilkins Centre for Molecular Biodiscovery The University of Auckland Auckland New Zealand; ^5^ Department of Medicine University of Calgary NB Calgary Alberta Canada

**Keywords:** bioelectronics, diagnostics, functional gastrointestinal disorders, gastric motility

## Abstract

**Background:**

Disorders of gastric function are highly prevalent, but diagnosis often remains symptom‐based and inconclusive. Body surface gastric mapping is an emerging diagnostic solution, but current approaches lack scalability and are cumbersome and clinically impractical. We present a novel scalable system for non‐invasively mapping gastric electrophysiology in high‐resolution (HR) at the body surface.

**Methods:**

The system comprises a custom‐designed stretchable high‐resolution “peel‐and‐stick” sensor array (8 × 8 pre‐gelled Ag/AgCl electrodes at 2 cm spacing; area 225 cm^2^), wearable data logger with custom electronics incorporating bioamplifier chips, accelerometer and Bluetooth synchronized in real‐time to an App with cloud connectivity. Automated algorithms filter and extract HR biomarkers including propagation (phase) mapping. The system was tested in a cohort of 24 healthy subjects to define reliability and characterize features of normal gastric activity (30 m fasting, standardized meal, and 4 h postprandial).

**Key Results:**

Gastric mapping was successfully achieved non‐invasively in all cases (16 male; 8 female; aged 20–73 years; BMI 24.2 ± 3.5). In all subjects, gastric electrophysiology and meal responses were successfully captured and quantified non‐invasively (mean frequency 2.9 ± 0.3 cycles per minute; peak amplitude at mean 60 m postprandially with return to baseline in <4 h). Spatiotemporal mapping showed regular and consistent wave activity of mean direction 182.7° ± 73 (74.7% antegrade, 7.8% retrograde, 17.5% indeterminate).

**Conclusions and Inferences:**

BSGM is a new diagnostic tool for assessing gastric function that is scalable and ready for clinical applications, offering several biomarkers that are improved or new to gastroenterology practice.

## Graphical abstract

A novel scalable electrode array and system is presented that employs high‐resolution flexible electronics attached to a portable data logger, capable of non‐invasively mapping gastric motility accurately from the surface of the abdominal skin.

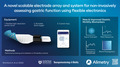

**Key Points**

- Chronic gastric symptoms affect up to 10% of adults, are increasing in prevalence, and impart a vast quality of life and cost burden. However, accurately defining, differentiating, and subtyping these overlapping conditions remains a critical problem in gastroenterology, owing to a lack of reliable, accessible objective diagnostic tests.
- This study presents a novel, scalable, system for body surface gastric mapping which accurately measures the stomach’s electrical activity and presents novel spectral and spatial biomarkers including high‐resolution spatiotemporal mapping of gastric slow waves from the skin surface.
- This body surface gastric mapping system has now validated and ready for clinical applications.

## 1 INTRODUCTION

Chronic gastric symptoms affect up to 10% of adults, are increasing in prevalence, and impart a vast quality of life and cost burden.^1^, ^2^, ^3^, ^4^ These symptoms encompass early satiation, excessive postprandial fullness, epigastric pain and burning, nausea and vomiting, and are clinically recognized in functional dyspepsia, chronic nausea and vomiting syndromes, and gastroparesis.^5^ However, accurately defining, differentiating and subtyping these overlapping conditions remains an important problem in gastroenterology, owing to a lack of objective diagnostic tests. The only widely available test of gastric function is measurement of gastric emptying, which is used to define gastroparesis, but is controversial due to its limited specificity, weak correlation with symptoms, and inconsistency over time.^4^, ^6^ There is a need for new tests of gastric function, and an ideal test would offer actionable biomarkers while being safe, non‐invasive, scalable and accessible, and cost‐efficient.^7^

A century ago, Walter C. Alvarez introduced electrogastrography (EGG) as a non‐invasive diagnostic tool for gastric function.^8^ EGG measures the bioelectrical slow waves that coordinate gastric motility, and also registers gastric contractile activity through an increase in signal power.^9^, ^10^ A substantial literature has been generated to show that EGG abnormalities are consistently prevalent in patients with gastric symptoms,^11^, ^12^, ^13^ yet EGG did not achieve common clinical adoption. Limitations to EGG include its focus on frequency as the predominant measure of abnormality, sensitivity to noise that could lead to misinterpretation of data, and an inability to account for a wide variability in gastric anatomical position.^14^, ^15^

Recently, body surface gastric mapping (BSGM or high‐resolution EGG) has been proposed as a novel diagnostic method to overcome the limitations of EGG, by employing a dense grid of electrodes to measure and map gastric activity in high‐resolution at the epigastrium.^14^, ^16^, ^17^ The convergence of an improved physiological understanding of gastric slow wave patterns in health vs disease states,^18^ modern amplifiers,^19^ and filters that more robustly discriminate gastric activity from noise,^20^, ^21^ have revitalized interest in the clinical potential of gastric electrophysiology.^14^ Recent studies have shown that BSGM can identify novel biomarkers such as slow wave direction or pattern that may offer superior symptom correlations compared to traditional EGG and gastric emptying testing.^16^, ^17^

Until now, the use of BSGM has been restricted to research applications. This is because the test has been impractical, involving the time‐consuming placement of many individual electrodes and managing the associated cable bundle, which is also a cleaning and disinfection barrier to clinical use. In addition, BSGM has required highly expensive specialized acquisition systems restricted to investigational use, and complex analysis approaches requiring substantial signal processing expertise. In this work, we developed a novel non‐invasive clinical solution for BSGM that overcomes these problems through the application of flexible electronics and miniaturized wearable circuitry, together with advances in automated data processing and visualization. The novel BSGM system and method was subjected to bench‐top verification followed by an experimental evaluation in healthy volunteers to demonstrate its reliability in generating improved or new clinical biomarkers of human gastric function, including robust frequency and power spectra, the direction and pattern of wave travel, and meal response metrics.

## 2 MATERIALS AND METHODS

### 2.1 System overview

**FIGURE 1:**
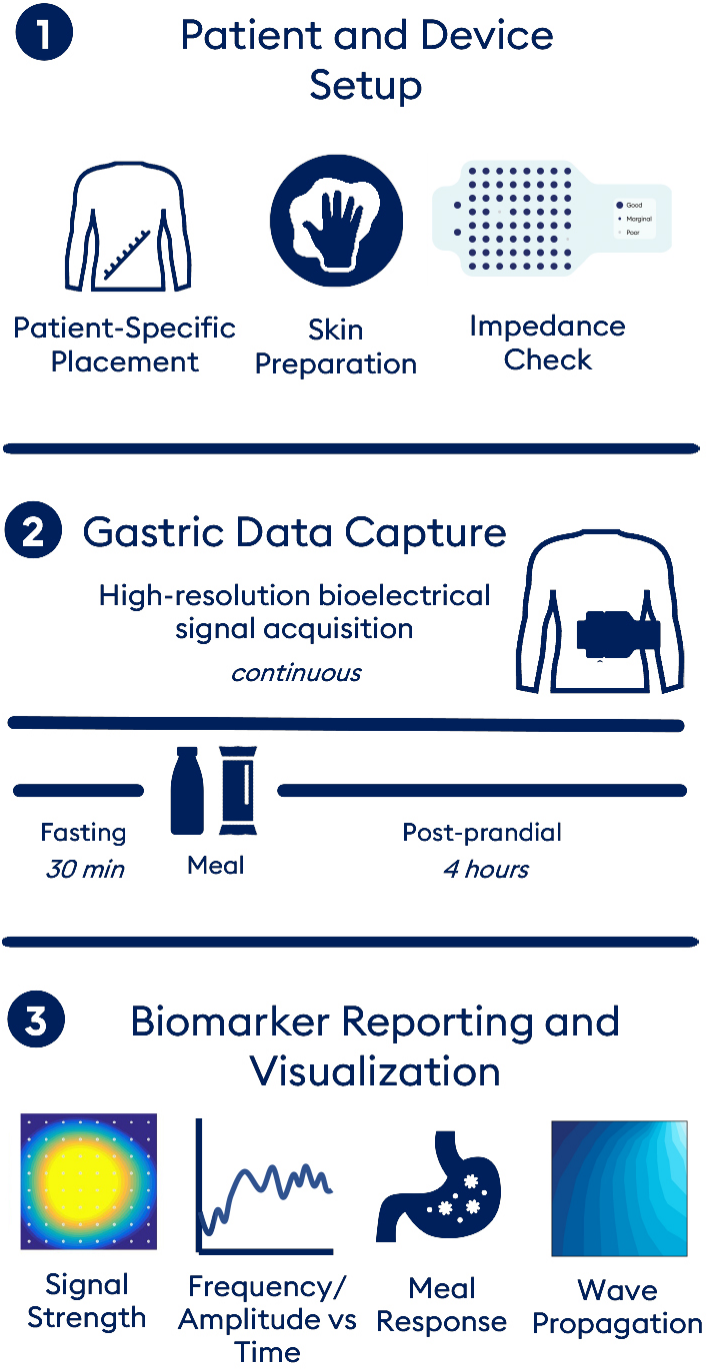
Body Surface Gastric Mapping (BSGM) workflow. (1) Setup includes placement of the sensor array personalized by measurements between anatomic landmarks, skin preparation with conductive gel, and a signal check using live impedance data displayed on the companion App. (2) Gastric activity is captured in HR continuously throughout the recording period (30 m fasted, standardized meal, up to 4 h postprandially). (3) A report of gastric activity is generated following automated signal processing and analyses. This includes a heat map used to infer gastric position by spatial distribution of amplitude, traditional gastric biomarkers including frequency and amplitude, along with novel meal response and spatial wave propagation biomarkers

**FIGURE 2:**
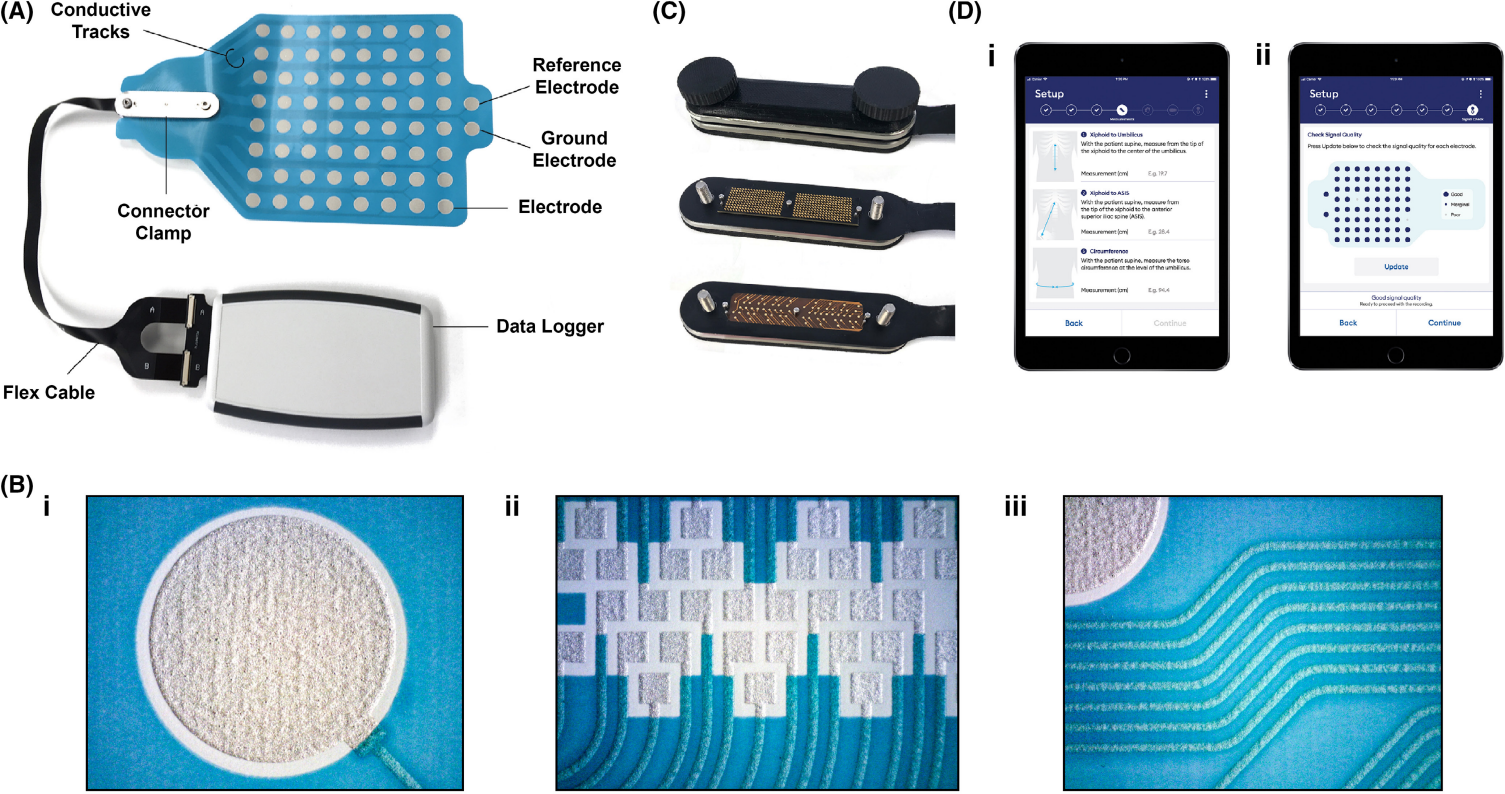
Components of Body Surface Gastric Mapping (BSGM) system. (A) Assembled BSGM system including sensor array, connector clamp, flexible printed circuit cable, and wearable data logger. (B) Close‐up of sensor array mating panel, demonstrating convergence of all 64 conductive tracks. This is opposed with the cable using the connector clamp shown in (C): thumb screws are loosened and the top piece removed to expose the connector piece (middle), which facilitates secure connection between the mating panel and the connector cable (bottom). (D) Companion App used to register test and participant details, customize recording variables, and guide setup of the sensor array and data logger. (D. i) Measurement input interface, where distances between the xiphoid and umbilicus, xiphoid and anterior superior iliac spine (ASIS), and abdominal circumference are recorded to guide personalized array positioning. (D. ii) Signal quality check shown on the App. The size and color of each electrode button represents the impedance measured for each channel

The novel BSGM system was designed to accommodate a clinical testing framework comprising a 30 min fasting baseline, consumption of a standardized meal, and up to 4 h of postprandial testing (Figure 1). This timeframe was chosen to reflect the typical period of the gastric meal response.^22^ The BSGM system (overviewed in Figure 2) is comprised of a flexible and conformable pre‐gelled “peel‐and‐stick” high‐resolution (HR) sensor array, an ambulatory data logger with custom electronics specifically tuned for gastric bioelectrical data, and a native iOS App with HIPAA‐compliant cloud connectivity and Bluetooth 5.0 synchronization to the data logger. The App guides the user through test setup, including user‐specific algorithm guided array positioning, requests symptom data from the subject during testing according to a validated design, and manages data transfers. Automated algorithms were also developed for filtering and extracting and visualizing clinical biomarkers. Each of these components are discussed in further detail below.

#### 2.1.1 Sensor array

**FIGURE 3:**
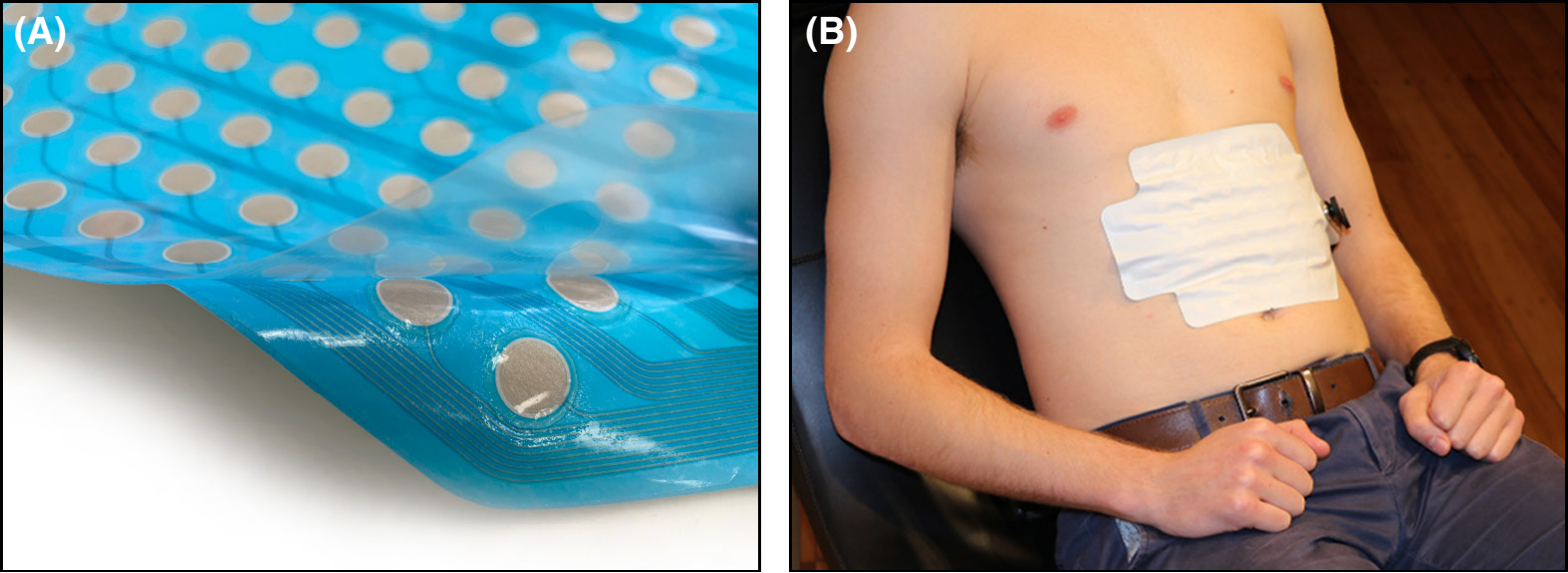
High‐resolution sensor array (8 × 8 pre‐gelled Ag/AgCl electrode grid; 2 cm spacing; 64 channels; area 225 cm^2^) printed upon a flexible TPU substrate, enabling comfort and optimal electrode contact across contours of the abdominal wall. (A) Backing layer partially peeled off, exposing adhesive layer and hydrogel discs. (B) Sensor array placed on a subject's epigastrium while reclined at 45 degrees

Gastric bioelectrical signals are of weak amplitude and signal strength diminishes exponentially as distance from the source increases,^23^ meaning that electrodes should be positioned directly over the stomach for capturing reliable data.^24^ An electrode sensor array was, therefore, designed to a size that was capable of achieving a position consistently overlying the stomach with high reliability when placed on the epigastrium (196 cm^2^; data based on a separate proprietary anatomical study). The sensor array (Figures 2A and 3A) was screen‐printed in layers on a 21 × 16 cm thermoplastic polyurethane (TPU) substrate. TPU was chosen for its ease of manufacturing, biocompatibility and high conformability, allowing comfortable adherence to the user's epigastrium (Figure 3B), including through a full range of movement without delaminating from the skin.

An 8 × 8 grid of electrodes (11 mm diameter, 20 mm center‐to‐center electrode spacing) with adjacent reference and ground electrodes was screen‐printed onto the TPU substrate using Ag/AgCl ink. Each electrode pad has an associated conductive track coalescing to a “mating panel” (Figure 2B). An insulating dielectric layer was then applied to coat the entire surface except for the electrode pads and mating panel (indicated by blue areas in Figure 2B). A conversion process was then undertaken (Figure 3A). First, an adhesive laminate was applied over the dielectric layer. Conductive hydrogel discs were then positioned on each Ag/AgCl electrode to ensure low‐impedance charge transfer at the skin interface.^25^ Finally, the adhesive and hydrogel layers were covered by a backing layer, which is peeled off immediately prior to application. The entire sensor array was mass‐fabricated with an automated process involving roll‐to‐roll screen‐printing, die‐cutting, and converting machinery, before individual packaging in moisture barrier foil pouches (Alimetry Ltd, New Zealand).

A total of 22 completed arrays underwent comprehensive testing to ensure adequate electrode quality and performance according to the ANSI/AAMI EC12:2000 Standard and a further 30 arrays underwent shelf life testing. Further details regarding the array testing procedure can be found in the Supplementary Methods.

#### 2.1.2 Connector

A compact board‐to‐board connector design was introduced that overcomes a key design challenge in the field of high‐resolution wearable electronics, being the attachment of a flexible array to a rigid circuit without the need for bulky connector attachments or cables. The sensor array interfaces with the data logger device by means of a custom flexible printed circuit cable and high density compression connector with a 0.2 mm thick PI stiffener on the back of the connector end, placed between a machined stainless‐steel clamp (Alimetry Ltd) (Figure 2C). The other end of the cable attaches to a zero‐insertion‐force connector on a custom mating PCB that allows repeatable connections to the data logger without additional tools.

#### 2.1.3 Data logger

An ambulatory data logger (Figure 2A) was designed and fabricated, employing all custom electronics and firmware, and housed within an off‐the‐shelf casing (Hammond Manufacturing, USA; 147 × 89 × 25 mm). Bioelectrical signals are recorded at 250 Hz, amplified and digitized by low‐noise programmable gain amplifiers with each input compared against a common reference electrode as shown on Figure 2A, to provide unipolar recordings for 64 channels. Data is stored on removable internal storage until uploaded to a HIPAA‐compliant cloud server via the App. An onboard accelerometer is used to record motion during the recording. Bluetooth connection with the App is maintained throughout the recording session, and to facilitate data upload. After a successful upload, data is securely deleted from the data logger in preparation for the subsequent test. After assembly, the data logger electronics underwent comprehensive electrical performance testing to ensure design criteria were met, as well as electromagnetic compatibility (EMC) testing. Further details regarding these testing methods are provided in the Supplementary Methods.

#### 2.1.4 App and array placement algorithm

The companion App was programmed in Swift v.5.1, being designed for use on an iPad mini (Apple, CA, USA). A password‐protected administration section allows the user to register the test and participant details, customize recording variables, and to guide setup. As noted above, reliable placement of the electrode array directly over the stomach location is an essential design requirement for reliable data capture in body surface gastric mapping. The App, therefore, further incorporated an array positioning algorithm taking into account guided measurements between xiphoid and umbilicus, xiphoid and anterior superior iliac spine (ASIS), and abdominal circumference (Figure 2Di).^26^ These measurements were used to calculate a patient‐specific array location with reference to the umbilicus, which is displayed to the user. Guided placement of the array by this algorithm, in conjunction with the chosen array size, aimed to reliably capture the gastric field within the recording electrode in the high majority of participants, by accounting for known anatomical variations (verified in a previous proprietary anatomical study).^26^ The App also undertakes an impedance check of the array prior to test initiation to ensure optimal data quality (Figure 2Dii).

### 2.2 Clinical and analytical methods

Ethical approval for the clinical studies was obtained from the Auckland Health Research Ethics Committee (AHREC, reference AH1130). This study focused on clinical evaluation of the novel system in 24 healthy subjects to demonstrate reliability for outputting each of the stated gastric biomarkers. Healthy subjects were 18 years or older with no known active GI symptoms or pathology, not meeting Rome IV criteria for a functional GI disorder, and not taking any medication known to affect gastrointestinal motility including anxiolytics and antidepressants. Additional exclusion criteria were any of the following: metabolic, neurogenic or endocrine disorder known to cause gastric dysmotility (including scleroderma, multiple sclerosis and hyperthyroidism), active GI infection, inflammatory bowel disease, previous gastric or esophageal surgery, history of GI malignancy, open abdominal wounds or abdominal skin not intact, fragile skin, allergy to adhesives and pregnancy. Additionally, those with body mass index (BMI) >35 were excluded, as data reliability is currently uncertain above this threshold.^24^

#### 2.2.1 Experimental protocol

Subjects were fasted for at least 8 h and were asked to not use nicotine or consume caffeine in the morning of their study. Measures of chronic gut symptoms were evaluated by the validated Patient Assessment of Gastrointestinal Disorders Symptom Severity Index (PAGI‐SYM)^27^ and Patient Assessment of Upper Gastrointestinal Disorders‐Quality of Life (PAGI‐QOL)^28^ questionnaires. The anterior abdomen was shaved if required and the skin prepped using a conductive gel (NuPrep; Weaver and Company, CO, USA). The novel flexible sensor array was then placed on the epigastrium, guided by the positioning algorithm. As per Figure 1, gastric activity was measured over a 30 min fasted period, followed by consumption of a standardized meal over 10 min, including a 232 kcal nutrient drink (230 ml Ensure; Abbott Nutrition, IL, USA) and an oatmeal energy bar (250 kcal with 5 g fat, 45 g carbohydrate, 10 g protein, 7 g fiber; Clif Bar & Company, CA, USA), and a further 4 h postprandial recording. Subjects remained reclined at 45 degrees for the entirety of the recording duration with their legs elevated in a comfortable position, and were instructed to limit movement and talking, avoid sleeping, and refrain from touching the array.^29^ At the end of the test, any adverse events were recorded and subjects were given a short questionnaire regarding system usability. Comfort during the test and any discomfort on removal of the array were assessed using electronic 100‐point visual analogue scales (0 ‐ “very uncomfortable” to 100 “very comfortable” for the test; and 0 “not painful” to 100 “most painful imaginable” for the array removal).

#### 2.2.2 Signal processing and analysis

Data collected using the BSGM device were processed using an automated proprietary algorithm that enabled filtering, biomarker outputs, and visualizations. In brief, each of the 64 channels were analyzed to first remove segments of significant artifact based on the methods of Gharibans et al.^21^ Further steps in the algorithm then generated the biomarkers of gastric function. Spatial heat maps were generated to show the predicted gastric location within the mapped field according to a power spectrum. Spectral analyses were performed using a composite of channels located centrally over the gastric position in the heat map, by a short‐time Fourier transform (4 minute windows with 75% overlap), visualized as frequency‐amplitude and amplitude‐time plots.^21^ Dominant frequency (cycles per minute; cpm), mean amplitude (μV), and variance in the dominant frequency were calculated for each participant and as summary statistics for the whole cohort. Meal response was characterized by the increase in the power of the spectral analysis after a meal (power ratio), and was calculated separately for the first 2 h postprandially (PR_2h_) and the entire postprandial period (PR_4h_).^30^ Average dominant frequency was calculated for the PR_2h_ phase, when signal power is high. The duration taken to return to a stable baseline was also calculated in each period with reference to the fasting period. The frequency‐amplitude spectrograms were also averaged, after normalizing amplitude for each participant, to define overall trends in the meal response power curve across the cohort. Mean amplitude was correlated against BMI.

Spatiotemporal metrics were derived for each subject using methods similar to those described by Gharibans et al.^16^, ^20^ Wave patterns were visualized as propagation animations,^23^ and their directionality was defined by manual classification. This was performed by five independent reviewers with conflicts resolved by consensus panel. Each reviewer visually assessed the animated data in 15 m epochs and classified these as antegrade, retrograde, indeterminate, or low‐amplitude noise, with the latter being excluded from subsequent percentage calculations. Summary data on wave directions were also computed via the algorithm and displayed as polar histograms.^16^, ^20^

#### 2.2.3 Statistical analysis

Normality was assessed by visual inspection of Q‐Q plots. Continuous independent normal variables were compared using Student's *t*‐test, and continuous independent non‐normal variables using the Mann–Whitney *U* test. More than two sets of continuous dependent normal variables were compared using the repeated measures ANOVA, with a Bonferroni post‐test correction applied. More than two sets of continuous dependent non‐normal variables were compared using Friedman's test, with a Dunn correction for multiple comparisons applied. Strength of association between variables was determined using Pearson's rank correlation coefficient (*r*). Sample size calculations for Array testing can be found in the Supplementary Methods. The statistical significance threshold was *p* < 0.05. All statistical analysis was performed using GraphPad Prism v.8 (GraphPad Software, CA, USA) and R version 4.0.3 (R Foundation for Statistical Computing, Vienna, Austria). Values are reported as the mean ± standard deviation (SD) unless stated otherwise.

## 3 RESULTS

### 3.1 Flexible array and data logger testing

The mass‐fabricated flexible arrays were tested to ensure the maximum Euclidean distance between ink layers was at most 1 mm, each hydrogel pad was only in contact with a singular Ag/AgCl electrode and that no adhesive layer overlapped with hydrogel pads. Based on testing of 22 arrays as determined by the a priori sample size calculation, all distances between Ag/AgCl and Ag layers for all electrodes were <1 mm; all distances between Ag/AgCl and dielectric layers for all electrodes were <1 mm; each hydrogel pad was in contact with exactly 1 Ag/AgCl electrode for all the electrodes on all the arrays and the adhesive layer did not overlap the hydrogel layer on any electrodes.

The array electrical testing met ANSI/AAMI EC12:2000 ECG Electrode Standards as detailed in Supplementary Results, together with the electrical performance testing of the data logger.

### 3.2 Clinical evaluation

Twenty‐four healthy subjects (16 male; 8 female) participated, of median age 28.5 (range 20–73 years). The mean BMI was 24.2 ± 3.5 kg/m^2^ (range 17.9–31.2), with 15 subjects of BMI < 25, and 9 of BMI ≥ 25). These healthy control subjects reported a minimal GI symptom burden (mean PAGI‐SYM 0.17 ± 0.33; mean GCSI 0.21 ± 0.40) and a high GI‐related quality of life (mean total PAGI‐QOL 4.84 ± 0.26).

### 3.3 Stomach localization, impedance and artifact

**FIGURE 4:**
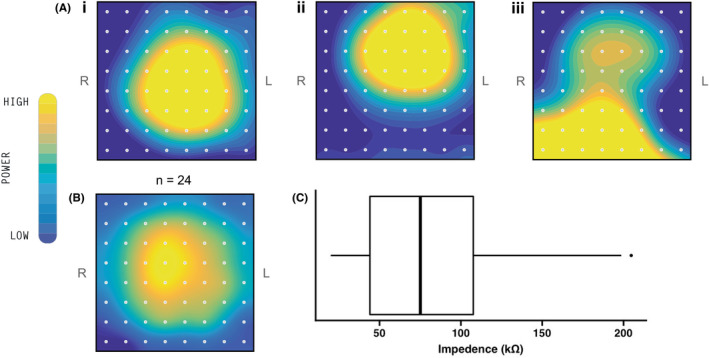
(A) Spatial heat maps for three participants, demonstrating the estimated signal power in the gastric range over the recording duration. Each electrode is represented by a gray circle, and the subject's left (L) and right (R) are indicated. Approximate stomach location can be inferred by the region of greatest amplitude (yellow). (B) Average heat map for all 24 participants. (C) Box and whisker plot of mean impedance for all 24 subjects

The sensor array positioning algorithm captured the area of high signal power from the stomach accurately in all 24 subjects. In 22/24 (92%) participants this area was located centrally within the mapped field, vs nearer to the edge of the mapped field in the remaining 2/24 (8%) (Figure 4A). The average heat map from all 24 recordings is shown in Figure 4B, confirming an overall average central position. The mean impedance was 84.1 kΩ (SEM 10.8 kΩ) (Figure 4C). The high majority of data were usable for analysis following automated removal of contamination by artifacts and subsequent signal recovery (mean 94.8 ± 4.8%).

### 3.4 Frequency, amplitude, and meal response

**FIGURE 5:**
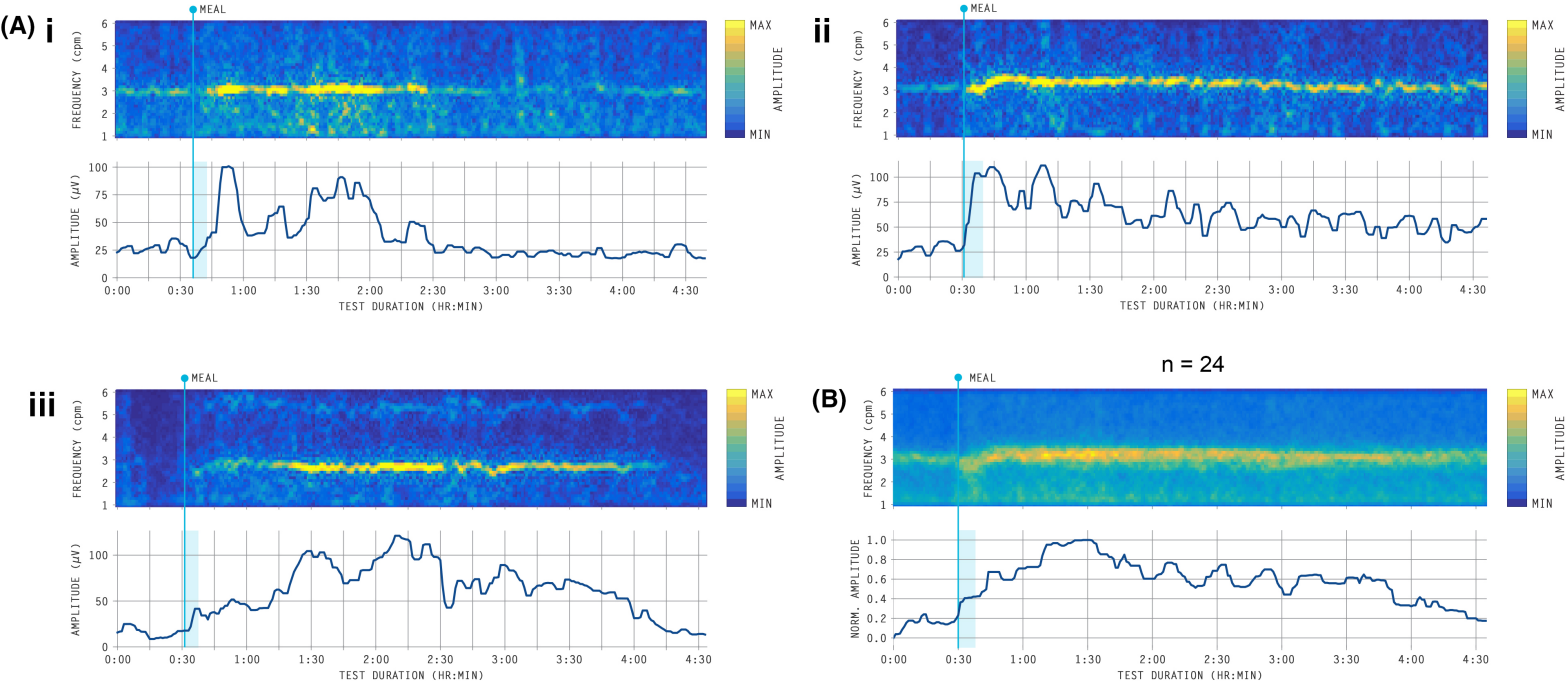
(A) Frequency‐amplitude spectrograms (top) and amplitude over time (bottom) for three subjects demonstrating features of the typical meal response. Start of meal time is marked by the blue line. Variability in the meal response was observed, with near‐immediate postprandial increases in amplitude (e.g., (i) and (ii)), or following a lag phase (e.g., [iii]). (B) Average frequency‐amplitude spectrogram for all 24 subjects, after normalization for amplitude. Amplitude steadily increased following the meal to reach a plateau at 1 h postprandially, before gradual return towards baseline (fasting) amplitude by the end of the 4 h postprandial period

**FIGURE 6:**
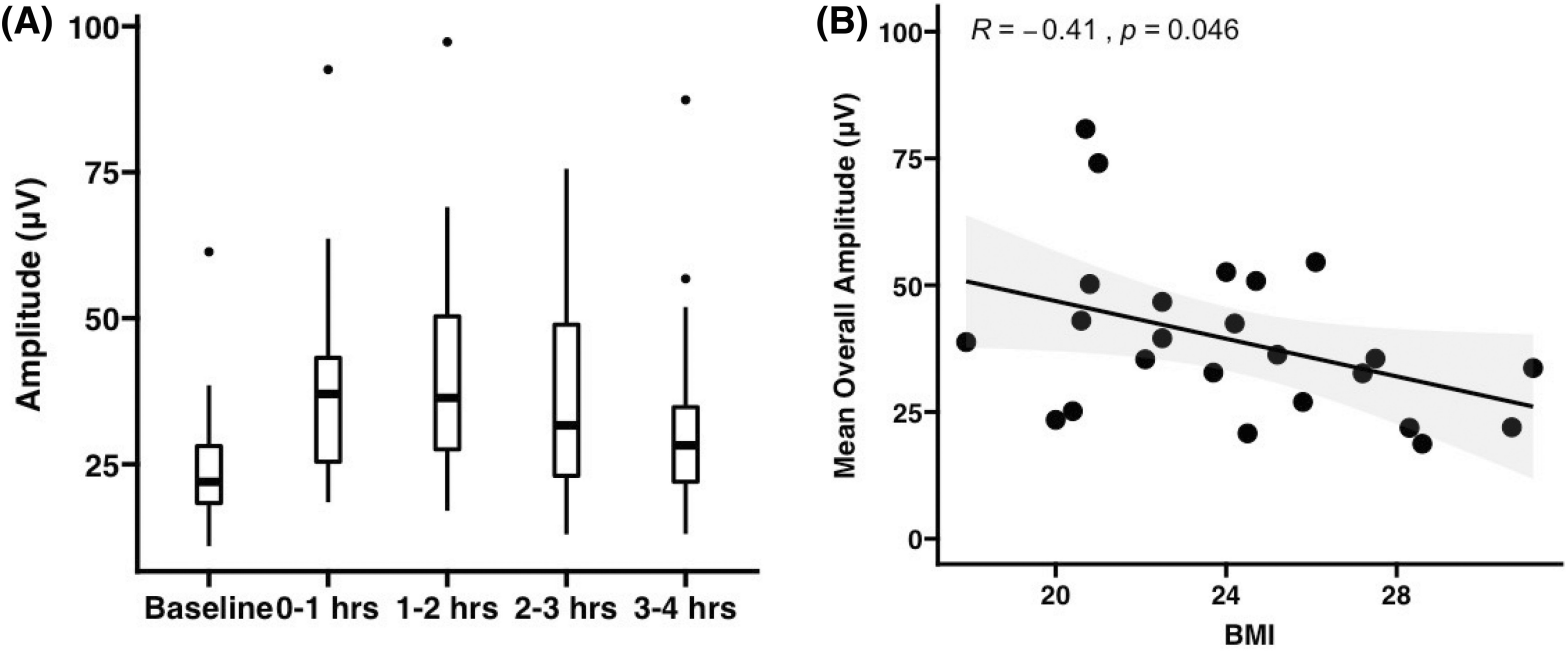
(A) Box and whisker plots of cohort mean amplitude over 1 h time periods. (B) Scatter plot of mean amplitude and BMI with a linear trend line applied

Clear dominant frequency bands were identified in all patients (e.g., Figure 5A,B). Mean dominant frequency was 2.9 cpm ± SD 0.3 (range 2.4–3.4 cpm), which was stable within participants (mean intra‐subject variability ± 0.43 cpm, range 0.10–0.83). Mean amplitude over the 4.5 h test duration ranged from 17.1 to 65.3 μV across the cohort (mean 35.2 ± SEM 2.7). The mean fasted amplitude was 24.2 ± SEM 2.2 μV, which was stable within participants (mean intra‐subject variability ± 3.7 μV, range 0.6–7.2). Mean amplitude significantly increased during the 0–2 h postprandial period (mean 39.1 μV ± SEM 3.2; *p* < 0.001 vs fasted period) and during the 2–4 h postprandial period was mean 34.3 μV ± SEM 3.2 (*p* = 0.0001 vs fasted period) (Figure 6A). Power ratios were: PR_2h_ 1.75 ± SD 0.90 and PR_4h_ 1.61 ± SD 0.73. Amplitude was negatively correlated with BMI (*r* = −0.41; *p* = 0.046; Figure 6B).

Three examples of meal responses are shown with frequency‐amplitude spectrograms in Figure 5A. These cases demonstrate the variability present in the time to maximum postprandial amplitude, which could occur nearly immediately after the meal (e.g., Figure 5Ai,ii), or following a lag phase (e.g., Figure 5Aiii). Figure 5A also demonstrates the variability in the length of the meal response (i.e., duration before return to baseline). The postprandial amplitude returned to baseline by 4 h postmeal in 21/24 subjects; the remaining subjects either did not show a substantial increase in power above baseline (*n* = 2; PR_2h_ 1.03 and 1.23), or amplitude did not return to baseline prior to the end of the test (*n* = 1; PR_2h_ 1.21 vs PR_3‐4h_ 2.72). The average frequency‐amplitude plot for all subjects is presented in Figure 5B. This data shows that, on‐average, amplitude steadily increased and peaked within 2 h after the meal, before gradually returning towards baseline fasted values by the end of the test.

### 3.5 Wave propagation profiling

**FIGURE 7:**
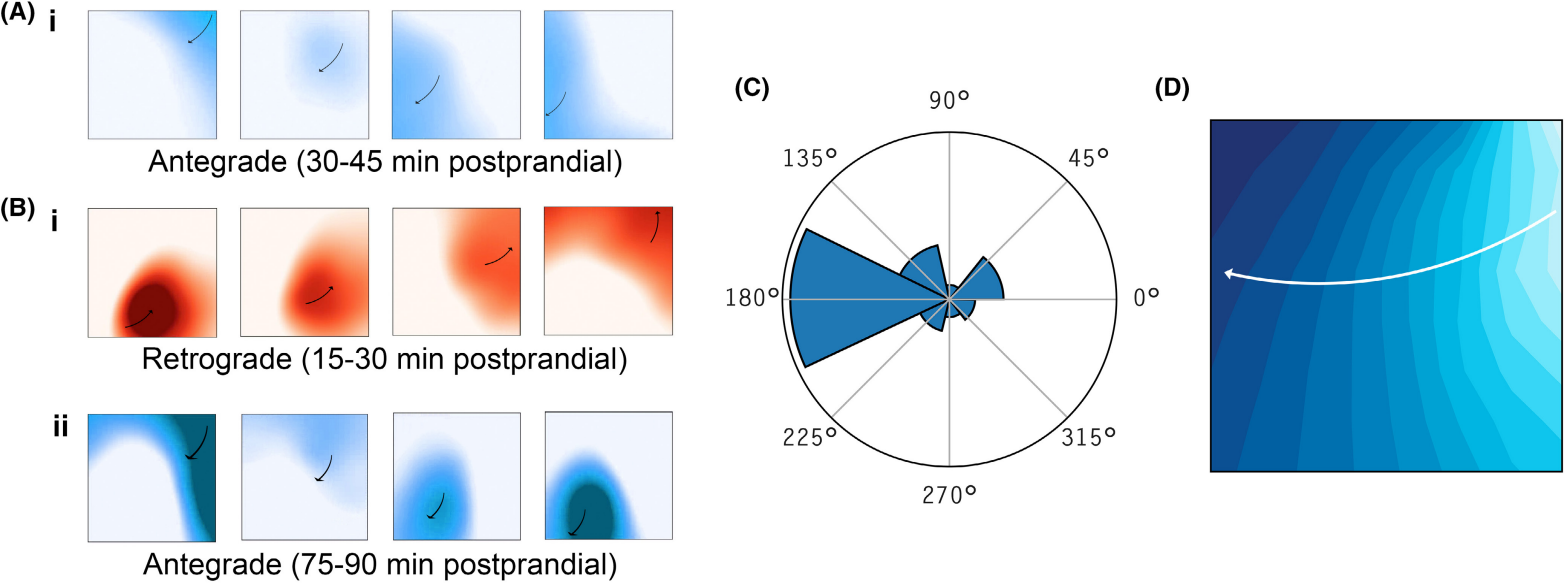
(A) Representative animations of postprandial periods of subject with antegrade activity; (B) representative animations of postprandial periods of subject with (i) retrograde activity immediately postprandially, which returned to antegrade activity soon after (ii); (C) Average polar histogram of all 24 subjects; (D) Average phase map of all 24 subjects

Spatiotemporal wave propagation profiling was performed in all 24 subjects. Example data are shown in Figure, with Figure 7A,I and Figure 7B,ii showing examples of typical antegrade propagation (see also Supplementary Animations). On classification, 74.7% of all subjects recordings were antegrade, 7.8% retrograde, and 17.5% were indeterminate due to apparent looping of the body surface dipole that precluded clear determination of direction.^31^ An example of retrograde activity is shown in Figure 7B,i. Retrograde waves were observed in 10/24 subjects, typically during the first 1.5 h postprandially, lasting median 22.5 min, and being sustained for >15 min in 7 subjects. Mean wave direction in the first 2 h postprandially was 182.7° ± 37 (Figure 7C). The average distribution of wave direction across all subjects is shown in the phase map in Figure 7D, demonstrating dominant antegrade propagation.

### 3.6 Safety

No participants had significant adverse events. One participant reported low‐grade itchiness related to the sensor array (resolution within 30 min), and another had moderate erythema of the epigastrium upon removal. Discomfort on removal of the sensor array was low for most subjects (mean 26.5 ± 22.2/100), while comfort during the test was high (mean 69.9 ± 27.1/100).

## 4 DISCUSSION

In this study we present a novel scalable flexible high‐resolution electrode array and complete clinical system for body surface gastric mapping (BSGM). First human data confirm that a dense field of gastric signal data was effectively captured in all subjects, enabling several biomarkers of gastric activity to be accurately characterized, including frequency and power spectra, meal response characteristics, and spatiotemporal metrics of wave propagation. These data demonstrate that the new system is a reliable non‐invasive tool for characterizing gastric electrophysiology at high‐resolution, offering a translational platform that is now ready for clinical applications. The test was well tolerated by all subjects, with no significant adverse events.

The BSGM system is shown to overcome several of the key technical limitations of EGG.^14^, ^32^ First, the combination of the large array size and a patient‐specific positioning system meant that the region of highest gastric signal power could be routinely and reliably captured within the mapped field. This is essential because amplitude falls exponentially as electrodes move off the stomach's location,^23^ which is highly variable.^26^, ^33^ Moreover, summating multiple signals from the region of highest gastric power substantially improves the accuracy of spectral analysis, reducing false positive data.^24^ Second, defining gastric propagation patterns is a valuable advance over EGG, because gastric dysrhythmias are now recognized as being spatially complex, with abnormal propagation patterns now known to occur at normal frequencies.^18^, ^34^ Third, the novel system integrates multiple technical improvements that together enhance signal‐to‐noise discrimination, which was a further key pitfall with EGG interpretation.^15^ These improvements included modern high‐fidelity bioamplifiers embedded in custom hardware and firmware specifically tuned to gastric parameters, and a conformable electrode array achieving low impedance at the skin interface.^21^

The solution introduced here also represents many advances over previous BSGM systems used in research applications. Gharbians et al and Somarajan et al. employed 5 × 5 grids of individually placed cardiac electrodes,^16^, ^17^, ^20^ while Bradshaw et al. employed a 4 × 4 grid.^35^ All of these systems required individual electrodes and cables, being cumbersome for clinical use and involving long setup times, as well as being difficult to reliably clean to hospital standards in an era of increasing concern regarding patient cross‐contamination. Individual electrode systems are also subject to inconsistent spacing and higher risk of cross‐talk. Our flexible and conformable mass‐fabricated electrode array overcomes these issues while obviating the need for prior imaging of the stomach's location due to its large size combined with an individualized positioning algorithm. The electrode density is much greater than previous approaches (including those employing flexible electronics^36^), enabled by the introduction of a screen‐printed solution, careful optimization of track routing, and a novel interposed clamp connector. This connector efficiently overcomes the problems of mating a stretchable array with a rigid circuit board and could, therefore, find utility in other fields of body surface electronics including cardiac, neural and skeletal muscle applications. Finally, the system also remains portable and user‐friendly, including an App to guide rapid standardized setup.

The presented system is fully scalable with arrays already being mass‐fabricated under ISO13485 standards, and is ready for clinical application. This is timely, because the objective assessment of gastric function is an area of current clinical concern. Gastric emptying measurement has recently fallen under renewed scrutiny after a prominent study reported that it did not adequately classify patients and was inconsistent over time.^6^ Gastric emptying correlates with symptoms when optimally performed, albeit weakly,^37^ but its role in guiding diagnosis and management remains controversial.^38^ Meanwhile, traditional forms of electrogastrography (EGG) are not currently recommended for clinical use,^39^ partly due to the limitations described above,^14^, ^32^ while other tests such as antroduodenal manometry and fundal accommodation testing generally remain restricted to niche applications and/or specialist centers.^40^, ^41^ New tests are needed that reference underlying pathophysiological mechanisms and provide actionable biomarkers in order to progress from symptom‐based diagnosis, diagnosis by exclusion, and trial‐and‐error therapy.^7^, ^38^ BSGM appears promising because it includes several biomarkers that hold promise independently or cumulatively, and which correlate with symptoms in emerging datasets,^16^ while being non‐invasive and accessible in any outpatient setting.^38^

The next step will be to apply the BSGM system in large cohorts of patients and controls to robustly assess clinical utility. The current data provides an indication of normal ranges for BSGM biomarkers, but it would be valuable to expand these to reference ranges, and then to evaluate whether these differ by demographics and with alternative meals. It was notable that a small proportion of gastric activity in our healthy subjects propagated in a retrograde direction (7.8% of mapped waves). Retrograde activation was not observed on previous invasive gastric mapping studies in fasted healthy subjects,^42^, ^43^ except for a single instance in an over‐inflated stomach,^34^ but has been reported postprandially in two other recent BSGM research studies,^16^, ^17^ and could relate to gastric distension.^44^ It, therefore, appears that retrograde wave behavior is part of the normal postprandial gastric repertoire, which would be an important physiological discovery,^45^ but this finding requires further scrutiny in dedicated studies before it can be accepted. Gastric electrophysiology has recently been confirmed by meta‐analyses to significantly deviate from healthy subjects in FD, CNVS, gastroesophageal reflux disease, and various pediatric disorders with traditional EGG,^11^, ^12^, ^13^, ^46^ indicating priority target groups for future clinical studies using BSGM. Additionally, studies suggest that dysrhythmias may also be implicated in a subset of postoperative gastric dysfunction.^47^, ^48^ Another important step will be to continue correlations of symptoms with specific biomarkers detected by BSGM, in order to further define pathophysiological features.^49^

This study is the first to suggest that a full meal response cycle can be robustly and routinely profiled non‐invasively at the body surface. In our healthy subjects, the postprandial power increase typically trended back to baseline by 3.5 h or earlier, with only one subject still showing elevated power at 4 h (Figure 5). The hypothesis is, therefore, presented that this meal response duration correlates with gastric emptying time. Historically, the typically 1 h EGG test has failed to correlate with gastric emptying,^32^ but this should now be re‐evaluated with 4 h BSGM in dedicated head‐to‐head testing. Gastric emptying is a function of caloric density and meal volume,^50^ and our test meal comprises a higher nutrient intake than a standard gastric emptying test (480 vs 255 kCal), which usually completes before 4 h.^22^ Smaller meals would, therefore, be warranted for gastric emptying comparisons.

Signal processing and analysis methods remain in ongoing development. A recent technical paper has validated the accuracy of BSGM in measuring the propagation direction of individual wavefronts at the body surface with direct reference to simultaneous high‐resolution serosal mapping, using similar techniques to those applied here.^51^ This study provides confidence that the technique is reliable, while also paving the way for future studies that provide more granular data on individual wave direction throughout entire recordings.^17^, ^35^ This step will also enable temporal correlations between symptom onset and retrograde wave patterns, in order to further elucidate the emerging clinical significance of retrograde gastric propagation.^52^ Further work to introduce and validate artifact identification schemes would be valuable. In addition, modeling and bench‐top studies also suggest that it may be possible in future to identify more complex wave patterns that occur in the stomachs of patients (e.g., colliding wavefronts, conduction blocks and re‐entrant activity),^53^ although this has not yet been validated experimentally. An additional limitation is that this study was performed in healthy subjects of normal weight, and the reliability of the system requires further validation in obese subjects. Previously, Gharibans et al have shown the ability to measure BSGM data up to a BMI of at least 35, which was, therefore, applied as the cut‐off here.^24^

In summary, our novel flexible electrode array and BSGM system provides a robust clinical solution for non‐invasively profiling gastric electrophysiology at the body surface. The system is scalable, validated, and ready for clinical applications, offering several biomarkers that are improved or new to gastroenterology practice.

## AUTHOR CONTRIBUTIONS

AG, GOG, and SC designed the device and research. AG, TH, DAC, SC, JW, and CK performed the studies. AG, TH, DAC, SC, CV, PD, SW, and JW analyzed the data. AG, YY, CA, and GOG provided supervision. AG, GOG, CV, TH, DAC, CA, SC, and YY wrote the paper and approved the final version for publication.

## CONFLICT OF INTEREST

AG, PD, CNA, and GO hold grants and intellectual property in the field of GI electrophysiology and are members of University of Auckland spin‐out companies: The Insides Company (GO), FlexiMap (PD), and Alimetry (AG, SC, YY, SW, JSTW, PD, CNA and GO). DAC, TCLH, and CV have no relevant conflicts to declare.

## PREVIOUS PRESENTATIONS

This work was presented at Digestive Diseases Week 2021: Gharibans, A., et al. (2021). “465 BODY SURFACE GASTRIC MAPPING: A NOVEL NON‐INVASIVE WEARABLE DIAGNOSTIC DEVICE FOR MEASUREMENT OF GASTRIC FUNCTION USING STRETCHABLE ELECTRONICS.” Gastroenterology 160(6): S‐95‐S‐96.

## Supporting information


Appendix S1

Video S1

Video S2

Video S3
