# Supplementary material for: A novel scalable electrode array and system for non‐invasively assessing gastric function using flexible electronics
Source: Neurogastroenterol Motil. 2022 Jun 14;35(2):e14418. doi: 10.1111/nmo.14418 (PMC10078595; doi:10.1111/nmo.14418)
Supplement: Supplementary file 3 — Video S2 [file NMO-35-0-s001.docx]

A novel scalable electrode array and system for non-invasively assessing gastric function using flexible electronics

Armen A. Gharibans^1,2,3^, Tommy C.L. Hayes^1^, Daniel A. Carson^1^, Stefan Calder^2^, Chris Varghese^1^, Peng Du^2,3^, Yaara Yarmut^2^, Stephen Waite^2^, Celia Keane^1,2^, Jon S.T. Woodhead^2,4^, Christopher N. Andrews^2,5^, Greg O’Grady^1,2,3^

**Supplementary Appendix:**

# Supplementary Methods

## Array testing

Array visual testing was designed to ensure adequate registration between ink layers, adequate separation of hydrogel pads, and avoidance of adhesive overlap with hydrogel pads. This was verified across 22 arrays using a digital microscope with measurement functionality (Dino-Lite, Taiwan).

The array’s electrical performance verification tests assessed according to the ANSI/AAMI EC12:2000 ECG Electrode Standards as follows:

- The average value of 10 Hz impedance for at least 12 electrode pairs from the sensor array connected gel-to-gel, at a level of impressed current not exceeding 100 μA peak-to-peak, shall not exceed 2 kilohms.
- The maximum value of 10 Hz impedance for at least 12 electrode pairs from the sensor array connected gel-to-gel shall not exceed 3 kilohms.
- After a 1-min stabilization period, a pair of electrodes from the sensor array connected gel-to-gel shall not exhibit an offset voltage greater than 100 mV.
- After a 1-min stabilization period, a pair of electrodes from the sensor array connected gel-to-gel shall not generate a voltage greater than 150 μV peak-to-peak in the passband (first-order frequency response) of 0.15 to 100 Hz, for a period of 5 min following the stabilization period.
- The observed DC voltage offset change across a pair of electrodes from the sensor array connected gel-to-gel shall not exceed 100 mV when the electrode pair is subjected to a continuous 200 nA DC current over a period of 8 hours.

In addition, each individual electrode on the Array was tested for electrical isolation from its neighboring electrodes by measuring the resistance between all neighboring electrodes.

Finally, accelerated shelf life testing was performed by placing arrays in a 60±3°C oven for 23-26 days (equivalent to 1 year real-time aging) and 46-49 days (equivalent to 2 year real-time aging), with 15 arrays used at each time point. Shelf life test samples underwent the following tests:

- visual inspection for discoloration, deformation, peeling off of layers compared to the original non-aged parts
- pouch burst testing according to ASTM F2054 (Standard Test Method for Burst Testing of Flexible Package Seals Using Internal Air Pressurization Within Restraining Plates)
- adhesion testing to verify adhesion to the skin
- testing no degradation of the readability of the labels when exposed to water and 70% Isopropanol/ 30% water solution
- electrical testing in accordance with ANSI/AAMI EC12:2000 ECG Electrode Standards.

### Array testing sample size justification

The sample size was determined based on the non-parametric Binomial distribution for attribute data, e.g., pass/fail or numerical results to fall within specification limits (Cheng S, Kupfer K, Dixon M, Shammas S. Optimized sampling design and rationale for verification and validation[^54^](https://paperpile.com/c/Jf4nl1/Abxi). The following formula was used from the Binomial distribution when zero failures occur,

$$n= \frac{ln(1-C)}{ln(R_{L})}$$

where, n denotes sample size, C denotes confidence and RL denotes reliability. Based on a confidence of 90% and reliability of 90%, 22 array samples were tested.

## Data logger testing

The data logger’s electrical performance ensured testing to meet the following performance requirements (adapted from IEC 60601-2:47 - Particular requirements for the basic safety and essential performance of ambulatory electrocardiographic systems):

- That internal noise referred to the input shall not exceed 50 μV p-v when all inputs are connected together;
- The amplitude response to sinusoidal signals within the frequency range 0.01 Hz to 0.25 Hz shall be between +3 dB and -3 dB of the response to 0.05 Hz;
- The channel to channel skew of the recorded electrophysiology data shall be less than ± 20 ms and that the output of the data logger shall be reproduced with a maximum amplitude error of ± 1% compared to a test signal at 0.05 Hz and 1 Vp-p;
- The gain change of the data logger shall not exceed 4% over a 5 hour period (in stable ambient conditions) and that the overall error in the data logger timing accuracy for collecting and storing electrophysiology data over 5 hours shall not exceed 5 seconds;
- The crosstalk between the channels of the data logger shall not produce in any channel an output referred to input greater than 5%.

Electromagnetic compatibility (EMC) testing was also performed according IEC 60601-1-2:2014 standard during recording and charging modes to ensure the data logger safety and performance in the presence of electromagnetic disturbances, and to test that the electromagnetic disturbances emitted by the device are within the specified limits.

**Supplementary Results**

The array electrical testing met ANSI/AAMI EC12:2000 ECG Electrode Standards. Accordingly, electrical testing of a random pair of electrodes across 22 arrays demonstrated that the average impedance across all 22 electrode pairs was 0.467 kΩ (none exceeding 3 kΩ), maximum DC voltage offset was less than 100 mV throughout 8 hours of testing (maximum 15.2 mV), and maximum peak-to-peak (p-p) noise was less than 150 μV throughout random 5 minute periods at a random electrode pair from 22 arrays (maximum 22.8 µV). The resistance between all neighboring electrodes on all arrays was greater than 1 MΩ, indicating sufficient electrode isolation to assess gastric propagation patterns. Array shelf life testing was performed on 30 additional arrays tested at both accelerated 1 and 2 year periods, confirming at least a 2 year shelf-life. The array therefore met the required standards of production quality.

## Data logger testing

Electrical performance testing of the data logger that internal noise referred to inputs did not exceed 1 μV p-v when all inputs were connected together and the amplitude response between the range 0.01 Hz to 0.25 Hz was between 99.3%-100% relative to the amplitude response at 0.05 Hz across 3 data loggers (**Fig. S1**). The amplitude range measured by all 64 channels for a 1000 µVp-p 0.05 Hz test input signal was between 1000.8 µV and 1004.4 µV. The channel to channel skew was 0 ms between all channels; all results were within the ± 1% acceptable range (i.e., between 990 µV to 1010 µV). The average amplitude for all channels throughout the entire 5 hour test was 1001.2 µVp-p, 1001.7 µVp-p, and 1001.6 µVp-p for each Data Logger respectively. The frequency for all channels was 0.0500 Hz for each channel and maintained throughout the 5 hours. The resistance between every possible combination of inputs on all 3 Data Loggers was greater than 1 MΩ.

**Supplementary Figures**

**Figure S1:** Test signal input overlay of all 64 channels.

**
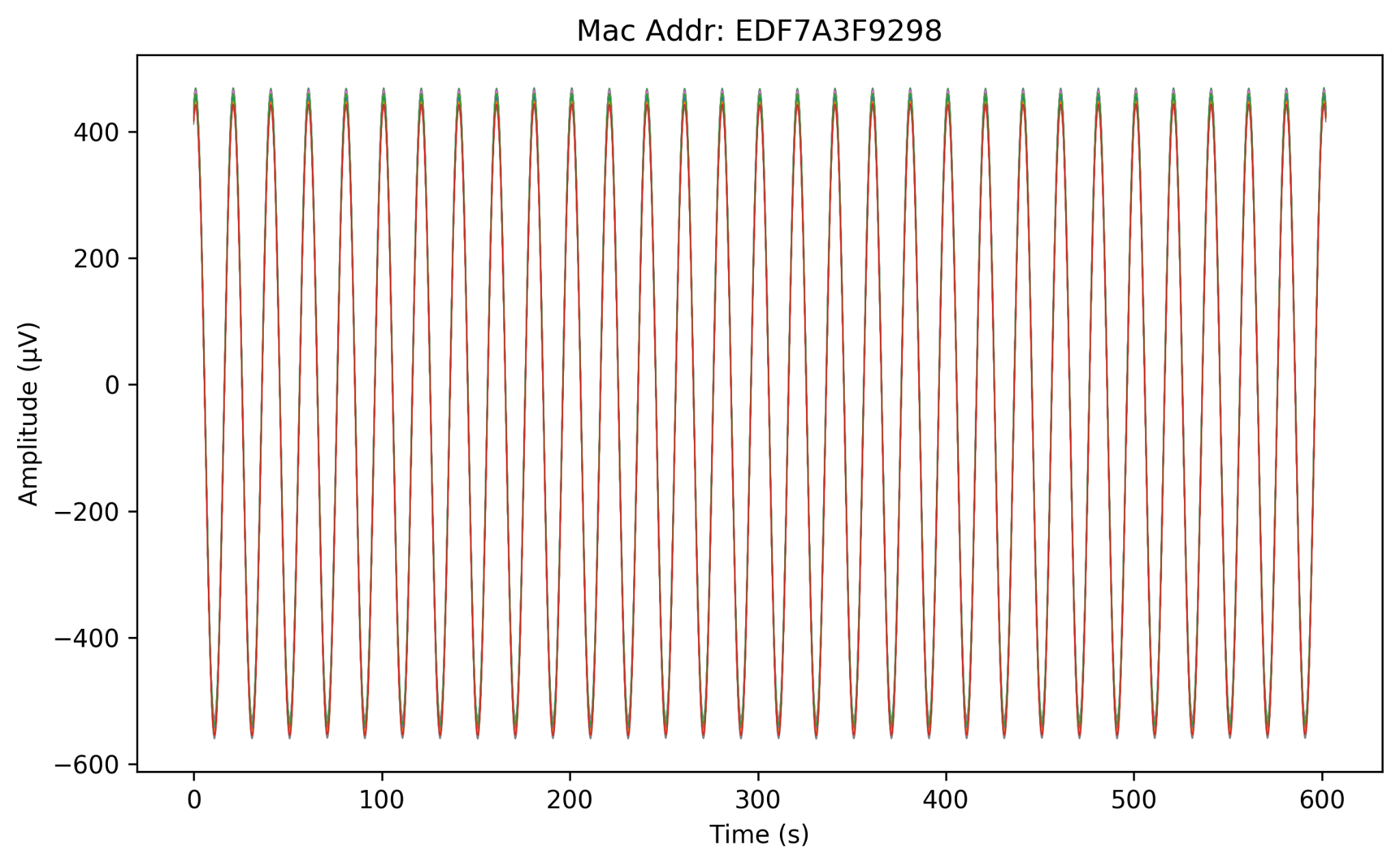
**

**Supplementary Animations**

Animated videos of subjects represented in Figure **7Ai, 7Bi and 7Bii**.
